# Molecular characterization of genomic breakpoints of *ALK* rearrangements in non‐small cell lung cancer

**DOI:** 10.1002/1878-0261.13348

**Published:** 2022-12-13

**Authors:** Zizong Wang, Yushuai Han, Houquan Tao, Mengxiang Xu, Zhengchuang Liu, Jianhua Zhu, Wei Li, Jie Ma, Zhifang Liu, Weiran Wang, Tonghui Ma

**Affiliations:** ^1^ Department of Thoracic Surgery The Affiliated Hospital of Qingdao University China; ^2^ Hangzhou Jichenjunchuang Medical Laboratory, Co., Ltd. China; ^3^ Key Laboratory of Gastroenterology of Zhejiang Province, Zhejiang Provincial People's Hospital People's Hospital of Hangzhou Medical College China; ^4^ Department of Pathology, Zhejiang Provincial People's Hospital People's Hospital of Hangzhou Medical College China; ^5^ Department of Clinical Pharmacology, Cheeloo College of Medicine, The Second Hospital Shandong University Jinan China

**Keywords:** *ALK* rearrangements, DNA‐based NGS, genomic breakpoints, non‐small cell lung cancer, RNA‐based NGS

## Abstract

*ALK* rearrangement is called the ‘diamond mutation’ in non‐small cell lung cancer (NSCLC). Accurately identifying patients who are candidates for ALK inhibitors is a key step in making clinical treatment decisions. In this study, a total of 783 *ALK* rearrangement‐positive NSCLC cases were identified by DNA‐based next‐generation sequencing (NGS), including 731 patients with *EML4‐ALK* and 52 patients with other *ALK* rearrangements. Diverse genomic breakpoints of *ALK* rearrangements were identified. Approximately 94.4% (739/783) of the cases carried *ALK* rearrangements with genomic breakpoints in the introns of *ALK* and its partner genes, and 2.8% (21/739) of these cases resulted in frameshift transcripts of *ALK*. Meanwhile, 5.6% (44/783) of the *ALK* rearrangement‐positive cases had breakpoints in the exons that would be expected to result in abnormal transcripts. RNA‐based NGS was performed to analyse the aberrant fusions at the transcript level. Some of these rearranged DNAs were not transcribed, and the others were fixed by some mechanisms so that the fusion kinase proteins could be expressed. Altogether, these findings emphasize that, when using DNA‐based NGS, functional RNA fusions should be confirmed in cases with uncommon/frameshift rearrangement by RNA‐based assays.

## Graphical abstract

Diverse genomic breakpoints of *ALK* rearrangements were identified by DNA‐based next‐generation sequencing (NGS) in non‐small cell lung cancer. RNA‐based NGS was performed to analyse the aberrant fusions. The actual transcript of uncommon *ALK* fusions may not match those predicted by the DNA genomic breakpoints. Altogether, when using DNA‐based NGS, functional RNA fusions should be confirmed in the cases with uncommon rearrangement by RNA‐based assays.

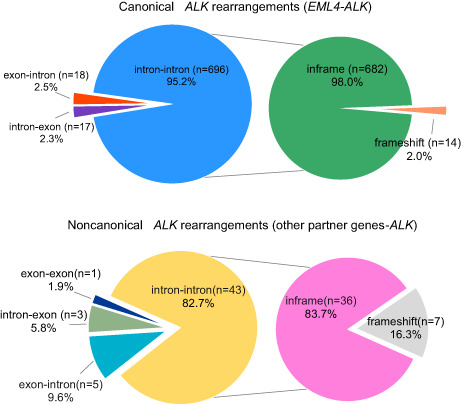

**Abbreviations**
ALK: anaplastic lymphoma kinase
EML4: echinoderm microtubule‐associated protein‐like 4
IGV: integrated genomics viewer
IHC: immunohistochemistry
KIF5B: kinesin family member 5B
KLC1: kinesin light chain 1
NGS: next‐generation sequence
NSCLC: non‐small‐cell lung cancer
QC: quality control
TKI: tyrosine kinase inhibitor
TPR: translocated promoter region

## 1 Introduction

Lung cancer is a major malignancy that threatens human life and health worldwide, with a high incidence and mortality in both male and female patients [1]. Non‐small cell lung cancer (NSCLC) is the main type of lung cancer, accounting for approximately 85% of cases, and it includes lung squamous cell carcinoma, lung adenocarcinoma and large cell lung carcinoma [2]. As a molecularly heterogeneous disease, multiple genetic alterations can drive the occurrence of NSCLC [3]. Approximately 3–7% of NSCLC patients harbour anaplastic lymphoma kinase gene (*ALK*) rearrangements [4, 5]. The wild‐type *ALK* gene encodes a transmembrane protein that is a classic receptor tyrosine kinase located on the cell membrane [6, 7, 8]. When the tyrosine kinase domain (exon 20 to exon 28) of ALK is retained in ALK‐containing fusion proteins, it results in oncogenic tyrosine kinases capable of driving oncogenesis through several downstream signalling pathways, including the RAS/MEK/ERK, PI3K and JAK/STAT pathways [6, 9]. Tyrosine kinase inhibitors (TKIs) represent a major milestone in the treatment of *ALK* rearrangement‐positive NSCLC patients, playing a crucial role in combating these oncogenic alterations [10, 11, 12].

Multiple methods have been developed to detect gene rearrangements/fusions in various clinical diagnostic settings [13, 14]. An assay utilizing DNA‐based next‐generation sequencing (NGS) has been applied frequently in recent years. Abundant types of *ALK* rearrangements are identified by DNA‐based NGS [15]. The most common partner gene for *ALK* is echinoderm microtubule‐associated protein‐like 4 (*EML4*) [16], and other noncanonical partner genes have been identified, such as kinesin family member 5B (*KIF5B*), kinesin light chain 1 (*KLC1*) and translocated promoter region (*TPR*) [17]. Previous studies have reported diverse genomic breakpoints of *ALK* rearrangements that occur in different regions (introns or exons) in NSCLC, and intronic breakpoint fusions usually result in in‐frame chimeric fusion transcripts/proteins [15, 18]. Multiple *ALK*‐fusion variants caused by variable genomic breakpoints have been reported with different sensitivities to ALK TKIs, especially in canonical *EML4‐ALK* fusions [19].

In theory, the potential pathogenicity of fusion variants ensures that the component of the kinase domain is in frame in the transcripts [20]. However, the predicted transcripts of some rearrangement types may be imprecise based on the coding sequence of the DNA. The potential unreliability of genomic breakpoints identified by DNA‐based NGS in predicting fusion transcripts has been proposed [15]. Therefore, the validation of *ALK* rearrangements detected at the DNA level, especially the uncommon genomic breakpoints of rearranged genes, needs to be constantly supplemented [21].

In this study, we retrospectively analysed the DNA molecular characteristics of *ALK* rearrangements in a local NSCLC database, and *ALK* rearrangements with noncanonical partner genes and uncommon genomic breakpoints were identified. To explore the actual transcripts of these rearrangements, which may result in abnormal transcripts, an RNA‐based NGS assay was performed. This study aimed to effectively and accurately determine the actual fusion status of the *ALK* gene in the context of specific *ALK* rearrangements.

## 2 Materials and methods

### 2.1 Patients and samples

From February 2018 to November 2021, a total of 783 lung cancer patient samples (718 tissues and 65 plasma fractions) were recruited from the Affiliated Hospital of Qingdao University, the Zhejiang Provincial People's Hospital and the Second Hospital of Shandong University, and these cases were detected as *ALK* rearrangement‐positive by DNA‐based NGS. In this study, *ALK* rearrangements retaining the 3′ ALK kinase domain were included and divided into canonical (*EML4‐ALK*) and noncanonical (other partner genes*‐ALK*) types. Their clinical characteristics were collected from their medical records and analysed. This study was conducted in accordance with the Declaration of Helsinki (as revised in 2013), and it was approved by the ethics committee of Zhejiang Provincial People's Hospital (No. QT2022218). The experiments were undertaken with the understanding and written consent of each subject.

### 2.2 DNA sample extraction and library construction

The sequencing methods have been described in earlier papers [22]. DNA samples from NSCLC patients were analysed using targeted deep sequencing with NGS technology. Genomic DNA was extracted from FFPE samples using a QIAamp DNA FFPE Tissue Kit (Qiagen, Valencia, CA, USA) following the manufacturer's instructions. Plasma cfDNA was extracted using a MagMAX Cell Free DNA Isolation Kit (Thermo Fisher Scientific, Waltham, MA, USA). DNA samples were quantified with the Qubit 2.0 Fluorometer using a Qubit dsDNA HS Assay kit (Life Technologies, Carlsbad, CA, USA) following the manufacturer's instructions. Genomic DNA from each FFPE sample was sheared into 150‐ to 200‐bp fragments using the M220 Focused‐ultrasonicator (Covaris, Woburn, MA, USA). Fragmented genomic DNA and cfDNA libraries were constructed with the KAPA HTP Library Preparation Kit (KAPA Biosystems, Wilmington, MA, USA) following the manufacturer's protocol. The concentration of DNA in the library was determined using the Qubit dsDNA HS assay kit.

### 2.3 Sequencing data analysis

DNA libraries were analysed using an OncoFocus panel (Genetron Health, Beijing, China), which includes 63 major lung cancer‐related genes. Quality control was undertaken on the raw sequencing data to remove the adapters and low‐quality regions using trimmomatic version 0.36 (Max Planck Institute of Molecular Plant Physiology, Potsdam, Germany). Local alignments of reads to the hg19 genome (GRch37) were carried out using the burrows–wheeler aligner tool (version 0.7.10) [23]. Somatic single nucleotide variants were retrieved using mutect (https://software.broadinstitute.org/cancer/cga/mutect) [24], somatic insertions and deletions were retrieved using strelka (https://github.com/Illumina/strelka) [25], and structural variations were determined using genefuse version 0.6.1 (https://github.com/OpenGene/GeneFuse) [26]. The variants were filtered and excluded with a population frequency over 0.1% based on guidelines by the Exome Aggregation Consortium. The remaining variants were annotated with Oncotator and Vep.

### 2.4 RNA‐based NGS

A Fusioncapture panel (Genetron Health, Beijing, China), which is a 395‐gene RNA panel, was used to identify gene fusions at the transcript level. Total RNA was isolated using the AllPrep DNA/RNA Mini Kit (Qiagen) and then reverse transcribed to cDNA using SuperScript III Reverse Transcriptase (Thermo Fisher Scientific, Waltham, MA, USA). The libraries were constructed with the KAPA HTP Library Preparation Kit (KAPA Biosystems) and subjected to Illumina HiSeq X‐Ten for paired‐end sequencing. Sequencing reads were mapped to a human reference genome (hg19) using hisat2‐2.0.5 (Johns Hopkins University School of Medicine, Baltimore, MD, USA). Gene fusions were identified using fusionmap [27].

### 2.5 Immunohistochemistry

The immunohistochemistry (IHC) assay has been described in the earlier studies [14, 28]. The IHC for ALK protein expression was performed on FFPE sections using a VENTANA ALK (Clone D5F3) CDx Kit and benchmark Ultra Immunostainer (Ventana Medical Systems, Inc., Tucson, AZ, USA, Cell Signaling Technology, Danvers, MA, USA) according to the manufacturer's instructions. The presence of granular cytoplasmic staining in the tumour cells (any percentage of positive tumour cells) was considered positive for ALK, while the absence of granular cytoplasmic staining in the tumour cells was considered negative for ALK.

### 2.6 Statistical analyses

The clinical characteristics of the study population were statistically analysed by the chi‐square test and Student's *t*‐test. A *P* value < 0.05 indicated statistical significance. Analyses and the data presentation were undertaken using ibm spss statistics 26.0 (IBM, Armonk, NY, USA) and graphpad prism 8.0.1 (GraphPad, La Jolla, CA, USA). The rearrangements and fusions were illustrated using Integrative Genomics Viewer, igv 2.11.4 (Broad Institute, Cambridge, MA, USA).

## 3 Results

### 3.1 Identification of *ALK* rearrangements in NSCLC patients

**Fig. 1:**
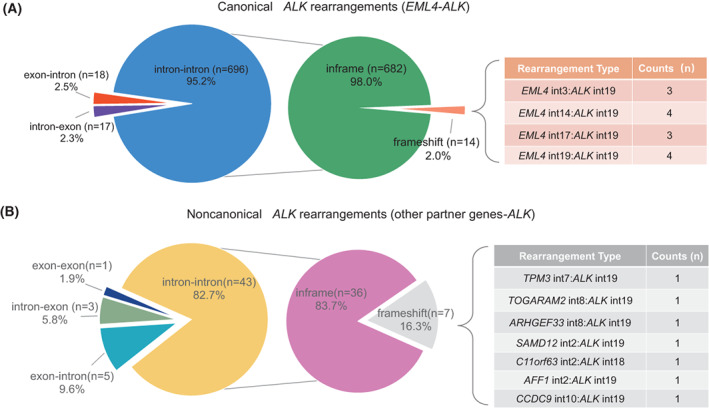
Categories of genomic breakpoints of *ALK* rearrangements by DNA‐based NGS (*n* = 783). (A) The distribution of canonical *ALK* rearrangements and the frequency of predicted transcript types in NSCLC patients with ‘intron–intron’ rearrangements. The table list the *EML4‐ALK* rearrangements whose predicted transcripts are frameshift. (B) The distribution of noncanonical *ALK* rearrangements and the frequency of the predicted transcript types with ‘intron–intron’ rearrangements. The table list the noncanonical *ALK* rearrangements whose predicted transcripts are frameshift.

In this study, a total of 783 *ALK* rearrangement‐positive cases were identified by DNA‐based NGS, including 731 cases with canonical *EML4‐ALK* rearrangements and 52 cases with noncanonical *ALK* rearrangements (Table 1). In these cases, with *EML4‐ALK*, 95.2% (696/731) of the cases carried rearrangements of *EML4* introns to incorporate *ALK* introns at the DNA level, 2.5% (18/731) of the cases were *EML4* exons rearranged to incorporate *ALK* introns, and 2.3% (17/731) of the cases were *EML4* introns rearranged to incorporate *ALK* exons (Fig. 1A). For noncanonical *ALK* rearrangements, the ‘intron–intron’, ‘exon–intron’, ‘intron–exon’ and ‘exon–exon’ types were identified in 82.7% (43/52), 9.6% (5/52), 5.8% (3/52) and 1.9% (1/52) of cases, respectively (Fig. 1B). In order for the *ALK*‐related fusion to be pathogenic, the *ALK* components have to remain in frame within the structure of the detected transcripts (*ALK* components that are out of frame would not be expected to be oncogenic because of the deletion of the kinase domain). We applied this logic to assess the DNA‐based NGS data and predicted chimeric transcripts of these fusion patterns. Thus, 2.0% (14/696) of the ‘intron to intron’ *EML4‐ALK* and 16.3% (7/43) of the ‘intron to intron’ noncanonical *ALK* rearrangements were predicted to be frameshifts with respect to the 3′ gene *ALK* (Fig. 1A,B). This prediction was based on the termination codon that appeared early due to the frameshift of the fusion transcript. The frameshift collection included four types of *EML4‐ALK* (intron 3 : intron 19, intron 19 : intron 19, intron 14 : intron 19 and intron 17 : intron 19) (Fig. 1A) and seven types of noncanonical *ALK* rearrangements (*TPM3* intron 7 : *ALK* intron 19, *TOGARAM2* intron 8 : *ALK* intron 19, *ARHGEF33* intron 8 : *ALK* intron 19, *SAMD12* intron 2 : *ALK* intron 19, *AFF1* intron 2 : *ALK* intron 19, *CCDC9* intron 10 : *ALK* intron 19 and *C11orf63* intron 2 : *ALK* intron 18) (Fig. 1B). Thus, we separated the *ALK* rearrangements into three categories: in‐frame, frameshift and exon breakpoints (‘exon to intron’, ‘intron to exon’ or ‘exon to exon’) (Table S1).

**Table 1:** Clinicopathologic feature in lung cancer patients with *ALK* rearrangements by DNA‐based NGS. ADC, adenocarcinoma; ex : int, exon to intron; int : ex, intron to exon; int : int, intron to intron.

Lung cancer feature	*ALK* rearrangements (*n* = 783)		*P* value
	*EML4‐ALK* (*n* = 731, 93.4%)	Other partner genes‐*ALK* (*n* = 52, 6.6%)	
Age, years			
Mean	54.5	56.4	0.244
Median	55	58	
Range	24–87	27–77	
Sex, *n*			
Male (%)	313 (42.8)	24 (46.2)	0.639
Female (%)	418 (57.2)	28 (53.8)	
Histotype, *n*			
ADC (%)	557 (76.2)	42 (80.8)	0.452
Non‐ADC (%)	11 (1.5)	0 (0)	
Unknown (%)	163 (22.3)	10 (19.2)	
Rearrangement type, *n*			
int : int (inframe) (%)	682 (93.3)	36 (69.2)	< 0.001
int : int (frameshift) (%)	14 (1.9)	7 (13.5)	< 0.001
ex : int (%)	18 (2.5)	5 (9.6)	0.011
int : ex (%)	17 (2.3)	3 (5.8)	0.286
ex : ex (%)	0 (0)	1 (1.9)	N/A

### 3.2 Characterization of the genomic breakpoints in the *ALK* rearrangements in the NSCLC patients

**Fig. 2:**
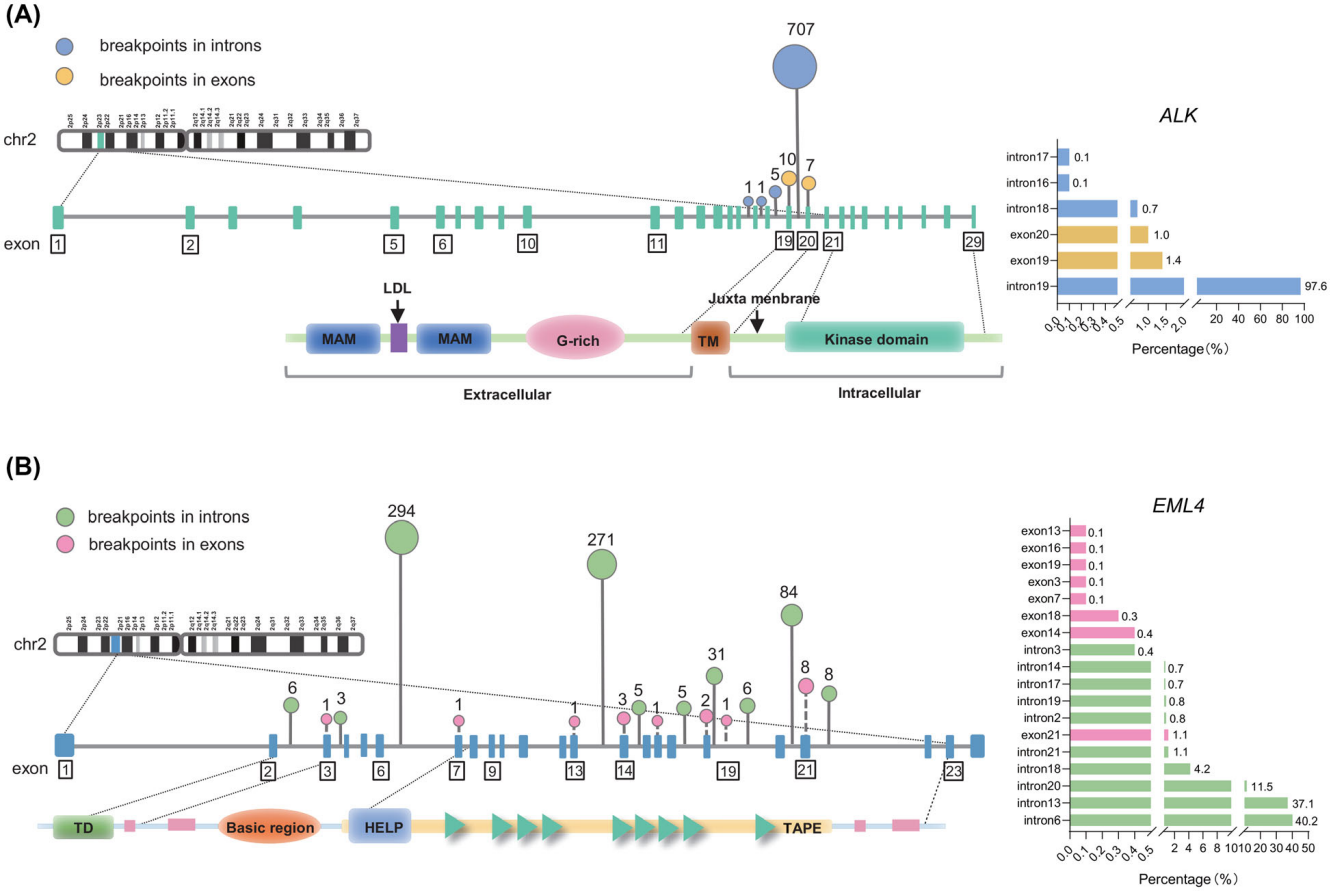
Protein domain structure and functional motifs of the *EML4* and *ALK* genes, as well as the distribution of the breakpoints of the *EML4‐ALK* rearrangements. (A) Protein structures of ALK and the distribution of the genomic breakpoints of *ALK*. Wild‐type ALK is a transmembrane protein and is a classical receptor tyrosine kinase located on the cell membrane. Its extracellular structures include two MAM (Meprin, A5 protein, and protein tyrosine phosphatase Mu) domains and one LDL, G‐rich, transmembrane (TM) kinase domain. The chart on the right shows the proportion of genomic breakpoints of *ALK*. Blue represents *ALK* introns, and yellow represents *ALK* exons. (B) Protein structures of EML4 and the distribution of genomic breakpoints of *EML4*. The N‐terminal coiled‐coil region forms a trimer structure (TD) that can combine with microtubules through the basic region, and the β‐propeller (TAPE) domain (composed of the hydrophobin motif (HELP) and the repeated WD40 domains) can bind to soluble tubulin. The chart on the right shows the proportion of genomic breakpoints of *EML4*. Green represents *EML4* introns, and pink represents *EML4* exons.

Based on our DNA NGS panel (covering all exons of *ALK*, as well as introns 16–20 of *ALK*), abundant information on the breakpoints was found. By analysing the genomic breakpoints of our *EML4‐ALK*‐positive samples, the breakpoints of *ALK* were found to be relatively concentrated and distributed in six of the regions. A total of 96.7% (707/731) of *ALK* breakpoint regions were located at intron 19, five cases were located at intron 18, and two cases were rearranged at intron 16 and intron 17. In addition, the *ALK* genomic breakpoints of 10 cases occurred in exon 19, and seven cases occurred in exon 20 (Fig. 2A). In contrast, the breakpoints of *EML4* were variable and distributed in 18 of the regions, most of which were in 10 intronic regions (713/731, 97.5%), the most common regions being intron 6 (294/731, 40.2%), intron 13 (271/731, 37.1%) and intron 20 (84/731, 11.5%). In addition, 2.5% (18/731) of the fusion breakpoints occurred in the exonic regions of *EML4* infrequently, including exon 3, exon 7, exon 13, exon 14, exon 16, exon 18, exon 19 and exon 21 (Fig. 2B). The distribution of common genomic breakpoints of *EML4* and *ALK* suggested common types of *EML4‐ALK* variants, including V1 (E13; A20), V2 (E20; A20) and V3 (E6; A20) (Table 2).

**Table 2:** Frequency of *EML4‐ALK* rearrangements based on DNA NGS. ex, exon; int, intron.

*EML4‐ALK* (*n* = 731)	Predicted RNA type (%)	DNA‐based NGS	Counts (%)
Intron : intron (*n* = 696)	Inframe^a^ (*n* = 682, 93.3%)	int13 : int19	267 (36.5)
		int20 : int19	82 (11.2)
		int6 : int19	282 (38.6)
		int2 : int19	5 (0.7)
		int18 : int19	31 (4.2)
		int6 : int18	4 (0.5)
		int6 : int16	1 (0.1)
		int21 : int19	8 (1.1)
		int6 : int17	1 (0.1)
		int13 : int18	1 (0.1)
	Frameshift^b^ (*n* = 14, 1.9%)	int14 : int19	4 (0.5)
		int17 : int19	4 (0.5)
		int3 : int19	3 (0.4)
		int19 : int19	3 (0.4)
Exon : intron (*n* = 18)	Exon breakpoints^b^ (*n* = 18, 2.5%)	ex7 : int19	1 (0.1)
		ex3 : int19	1 (0.1)
		ex21 : int19	8 (1.1)
		ex19 : int19	1 (0.1)
		ex18 : int19	2 (0.3)
		ex16 : int19	1 (0.1)
		ex14 : int19	3 (0.4)
		ex13 : int19	1 (0.1)
Intron : exon (*n* = 17)	Exon breakpoints^b^ (*n* = 17, 2.3%)	int6 : ex19	4 (0.5)
		int6 : ex20	2 (0.3)
		int2 : ex19	1 (0.1)
		int19 : ex19	1 (0.1)
		int19 : ex20	2 (0.3)
		int14 : ex20	1 (0.1)
		int13 : ex19	3 (0.4)
		int20 : ex19	1 (0.1)
		int17 : ex20	1 (0.1)
		int20 : ex20	1 (0.1)

a Common rearrangements.

b Uncommon rearrangements.

For noncanonical *ALK* rearrangements, *KIF5B*, *H1P1, DTCN1, KLC1*, *STRN*, *CLTC*, *CCDC9* and *PRKAR1A* were detected multiple times as partner genes, and the distribution of the genomic breakpoints of these partner genes was diverse (Table 3). Meanwhile, the genomic breakpoints of *ALK* were mainly distributed in intron 19 (43/52, 82.7%), which was similar to the canonical *EML4‐ALK* rearrangements, followed by intron 18 (5/52, 9.6%). Breakpoints in exons 17, 18, 19 and 20 of *ALK* accounted for one case each (Table 3).

**Table 3:** Catalog of noncanonical partner genes of *ALK* rearrangements based on DNA NGS. ex, exon; int, intron.

Rearrangements (inframe^a^, *n* = 36, 69.2%)	DNA‐based NGS	Counts (%)	Rearrangements (frameshift^b^, *n* = 7, 13.5%)	DNA‐based NGS	Counts (%)
*KIF5B‐ALK* (9)	int14 : int19	5 (9.6)	*TPM3‐ALK*	int7 : int19	1 (1.9)
	int17 : int19	3 (5.8)	*TOGARAM2‐ALK*	int8 : int19	1 (1.9)
	int11 : int19	1 (1.9)	*ARHGEF33‐ALK*	int8 : int19	1 (1.9)
*HIP1‐ALK* (8)	int28 : int19	6 (11.5)	*SAMD12‐ALK*	int2 : int19	1 (1.9)
	int19 : int19	1 (1.9)	*C11orf63‐ALK*	int2 : int18	1 (1.9)
	int21 : int19	1 (1.9)	*AFF1‐ALK*	int2 : int19	1 (1.9)
*DCTN1‐ALK* (3)	int26 : int19	2 (3.8)	*CCDC9‐ALK*	int10 : int19	1 (1.9)
	int27 : int19	1 (1.9)	**Rearrangements (exon breakpoints** ^**b**^ **, *n* = 9, 17.3%)**	**DNA‐based NGS**	**Counts (%)**
*KLC1‐ALK* (3)	int9 : int19	3 (5.8)			
*STRN‐ALK* (3)	int3 : int19	3 (5.8)	*PRR23C‐ALK*	ex1 : int19	1 (1.9)
*CLTC‐ALK* (2)	int31 : int18	2 (3.8)	*KIF5B‐ALK*	ex25 : int19	1 (1.9)
*PRKAR1A‐ALK* (2)	int10 : int19	1 (1.9)	*KAT6A‐ALK*	ex5 : int18	1 (1.9)
	int5 : int19	1 (1.9)	*CCDC9‐ALK*	ex10 : int19	1 (1.9)
*PRDM16‐ALK*	int1 : int19	1 (1.9)	*SQSTM1‐ALK*	ex5 : int19	1 (1.9)
*PLEKHH2‐ALK*	int6 : int19	1 (1.9)	*SFTPB‐ALK*	ex3 : ex17	1 (1.9)
*GCC2‐ALK*	int13 : int19	1 (1.9)	*PDIA6‐ALK*	int1 : ex18	1 (1.9)
*DHRS7‐ALK*	int1 : int19	1 (1.9)	*DCTN1‐ALK*	int13 : ex20	1 (1.9)
*CADPS‐ALK*	int11 : int18	1 (1.9)	*BRE‐ALK*	int11 : ex19	1 (1.9)
*ZFHX3‐ALK*	int5 : int19	1 (1.9)			

a Common rearrangements.

b Uncommon rearrangements.

### 3.3 Validation of the *ALK* frameshift rearrangement pattern by RNA‐based NGS

RNA‐based NGS was performed on nine stored samples as a frameshift cohort. Limited by the low‐quality RNA samples, two of the FFPE samples were not tested due to failure during the RNA quality control (QC) process. We finally detected seven qualified samples from available tissue, including four cases (#P2106140203, #P2011280013, #P2008100038 and #P1902170006) with *EML4‐ALK* and three cases (#P2007070051, #P2005010014 and #L‐2018‐00005429) with noncanonical *ALK* rearrangements (Table 4; Table S2). Furthermore, ALK‐IHC was performed on several available samples as a supplement validation of the NGS results, although there might be several mechanisms, such as *ALK* fusions, amplification and alternative transcription initiation of *ALK*, that can drive the overexpression of ALK and result in a positive IHC result [29, 30].

**Table 4:** Frameshift cohort of *ALK* rearrangements/fusions at DNA, RNA, protein levels. ex, exon; int, intron.

Patient ID	DNA‐based NGS	RNA‐based NGS	IHC
P2106140203	*EML4* int17 : *ALK* int19	*EML4* int17 : *ALK* ex20	Positive
P2103020235	*EML4* int14 : *ALK* int19	Fail	N/A
P2011280013	*EML4* int19 : *ALK* int19	*EML4* ex19 : *ALK* ex20	N/A
P2008100038	*EML4* int19 : *ALK* int19	Negative	Negative
P1902170006	*EML4* int3 : *ALK* int19	Negative	N/A
L‐2018‐00000747	*EML4* int14 : *ALK* int19	Fail	N/A
P2007070051	*TOGARAM2* int8 : *ALK* int19	*EML4* ex13 : *ALK* ex20	N/A
P2005010014	*SAMD12* int2 : *ALK* int19	*EML4* ex20 : *ALK* ex20	N/A
L‐2018‐00005429	*AFF1* int2 : *ALK* int19	*EML4* ex13 : *ALK* ex20	N/A

In two cases (#P2008100038 and #P1902170006), the *EML4‐ALK* fusions were negative at the transcript level despite positivity in DNA‐based NGS, and these results were confirmed by IGV (Fig. S1). We assumed that the rearranged genetic material of these two cases is not transcribed, and the ALK‐IHC results of case #P2008100038 showed that ALK protein expression was negative (Fig. S2C).

**Fig. 3:**
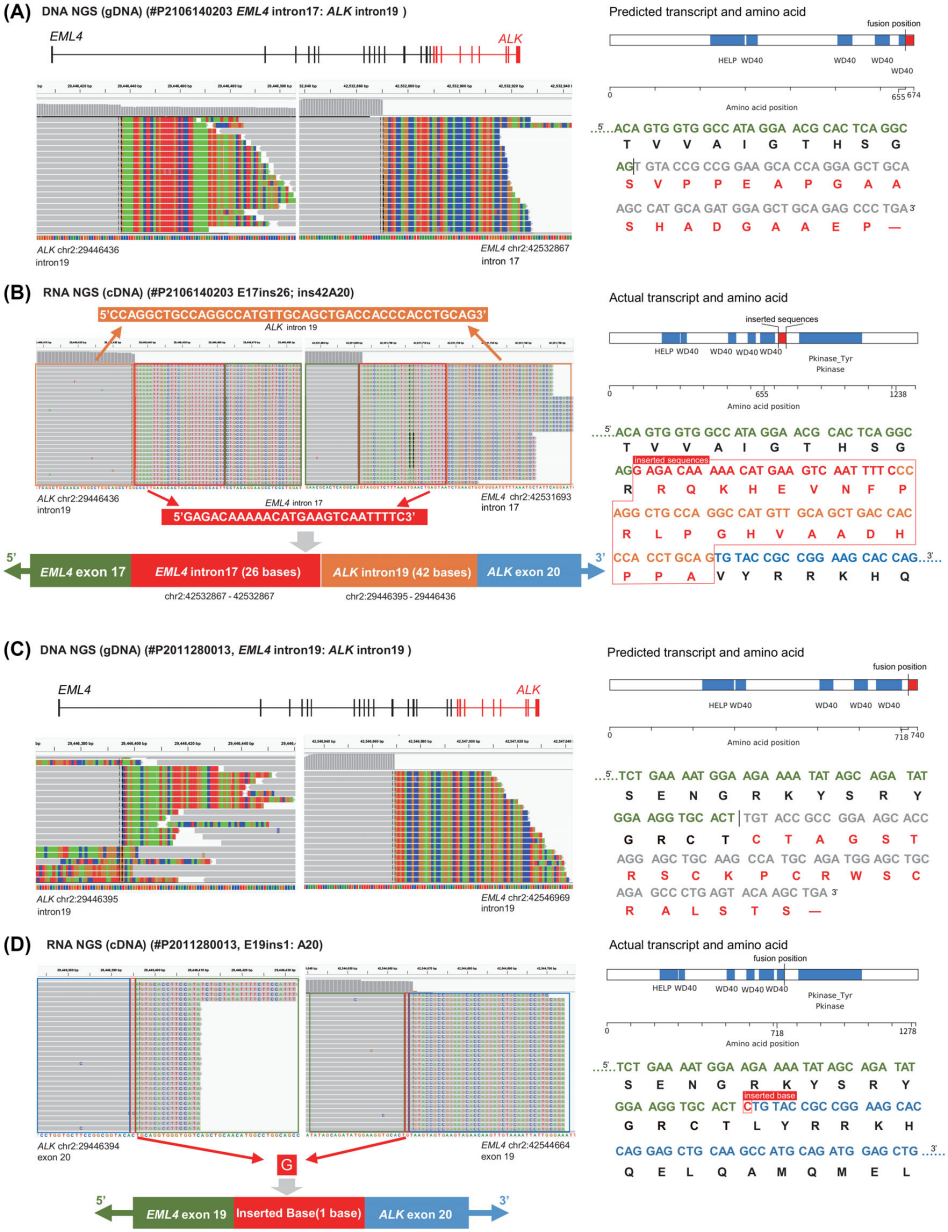
Integrative genomics viewer (IGV) screenshots, predicted or actual transcript and amino acid sequences of *EML4‐ALK* detected using DNA‐based NGS and RNA‐based NGS. Blue, green, red and orange blocks represent the ‘C’, ‘A’, ‘T’ and ‘G’ bases, respectively. (A, C) DNA‐NGS IGV screenshots and the predicted transcript and amino acid sequences of cases #P2106140203 and #P2011280013, respectively. (B, D) RNA‐NGS IGV screenshots and the actual transcript and amino acid sequences of cases #P2106140203 and #P2011280013, respectively.

Another 2 *EML4‐ALK‐*positive cases (#P2106140203 and #P2011280013) were positive for a fusion in both the RNA‐based and DNA‐based NGS assays. The predicted transcript of case #P2106140203 was exon 17 of *EML4* fused to exon 19 of *ALK* and it would not have been in frame (Fig. 3A). However, the actual transcript detected by the RNA‐based NGS assay of case #P2106140203 did not match the predicted transcript. The IGV showed a novel variant composed of a sequence derived from *ALK* intron 19 (42 adjacent nucleotides, 5′‐CCAGGCTGCCAGGCCATGTTGCAGCTGACCACCCACCTGCAG‐3′) and a sequence derived from *EML4* intron 17 (26 nonadjacent nucleotides, 5′‐GAGACAAAAACATGAAGTCAATTTTC‐3′) inserted between exon 17 of *EML4* and exon 20 of *ALK* (E17ins26; ins42A20) (Fig. 3B). In addition, the fusion type of case #P2011280013 was intron 19 of *EML4* fused to intron 19 of *ALK* at the genomic level, and the predicted transcript was not in frame (Fig. 3C). However, a novel variant (E19ins1; A20) with a nucleotide inserted between exon 19 of *EML4* and exon 20 of *ALK* was detected by RNA‐based NGS and it did not match the predicted transcript (Fig. 3D). The RNA NGS results of #P2106140203 and #P2011280013 suggested that these fusion types did not follow the conventional splicing signal in the exon–intron boundary but instead formed novel fusion variants. We performed IHC in the case (#P2106140203) with some remaining tissue and verified ALK protein expression positivity (Fig. S2D).

Moreover, three cases (#P2007070051: *TOGARAM*2 intron 8 : *ALK* intron 19; #P2005010014: *SAMD12* intron 2 : *ALK* intron 19; #L‐2018‐00005429: *AFF1* intron 2 : *ALK* intron 19) with noncanonical *ALK* rearrangements were detected by RNA‐based NGS, and the results showed *EML4‐ALK* fusions at the level of transcription (Table 4; Fig. S3A–C), indicating that the splicing alteration of the *ALK* frameshift rearrangements was different between *EML4‐ALK* and the noncanonical *ALK* rearrangements.

### 3.4 Validation of the *ALK* rearrangement patterns with genomic breakpoints at the exons

In contrast to conventional genomic breakpoints of gene fusions that are located at introns, some partial breakpoints that led to gene fusions observed in this study occurred at exons. We detected the sequence of the fusion transcripts by RNA‐based NGS from 13 available samples to explore the actual transcripts produced by the *ALK* rearrangements with genomic breakpoints located in the exons of *ALK* or its partner genes. Nine of the 13 samples were qualified by the RNA QC metric, including six cases (#P2006160041, #P2107150201, #P1911070042, #P2010230040, #P2009100057 and # P2008280124) with *EML4‐ALK* and three cases (#P2009120038, #P2004270003 and #P2003200071) with noncanonical *ALK* rearrangements (Table 5; Table S2).

**Table 5:** Genomic breakpoints in exons cohort of *ALK* rearrangements/fusions at DNA, RNA, protein levels. ex, exon; int, intron.

Patient ID	DNA‐based NGS	RNA‐based NGS	IHC
P2006160041	*EML4* ex3 : *ALK* int19	*EML4* ex2 : *ALK* ex20	N/A
P2107150201	*EML4* ex14 : *ALK* int19	*EML4* ex13 : *ALK* ex20	Positive
L‐2018‐00002291	*EML4* ex14 : *ALK* int19	Fail	Positive
P1901260022	*EML4* ex14 : *ALK* int19	Fail	N/A
P1911070042	*EML4* ex21 : *ALK* int19	*EML4* ex20 : *ALK* ex20	N/A
L‐2018‐00010529	*EML4* ex21 : *ALK* int19	Fail	Positive
P2010230040	*EML4* int6 : *ALK* ex19	*EML4* int6 : *ALK* ex20	N/A
P2009100057	*EML4* int13 : *ALK* ex19	*EML4* ex13 : *ALK* ex20	N/A
P2008280124	*EML4* int19 : *ALK* ex20	*EML4* ex19 : *ALK* ex20 (partial)	Positive
P2011280079	*EML4* int20 : *ALK* ex19	Fail	N/A
P2009120038	*PRR23C* ex1 : *ALK* int19	*KIF5B* ex17 : *ALK* ex20	N/A
P2004270003	*SQSTM1* ex5 : *ALK* int19	*SQSTM1* ex4 : *ALK* ex20	N/A
P2003200071	*SFTPB* ex2 : *ALK* ex17	*SFTPB* ex1 : *ALK* ex18	N/A

**Fig. 4:**
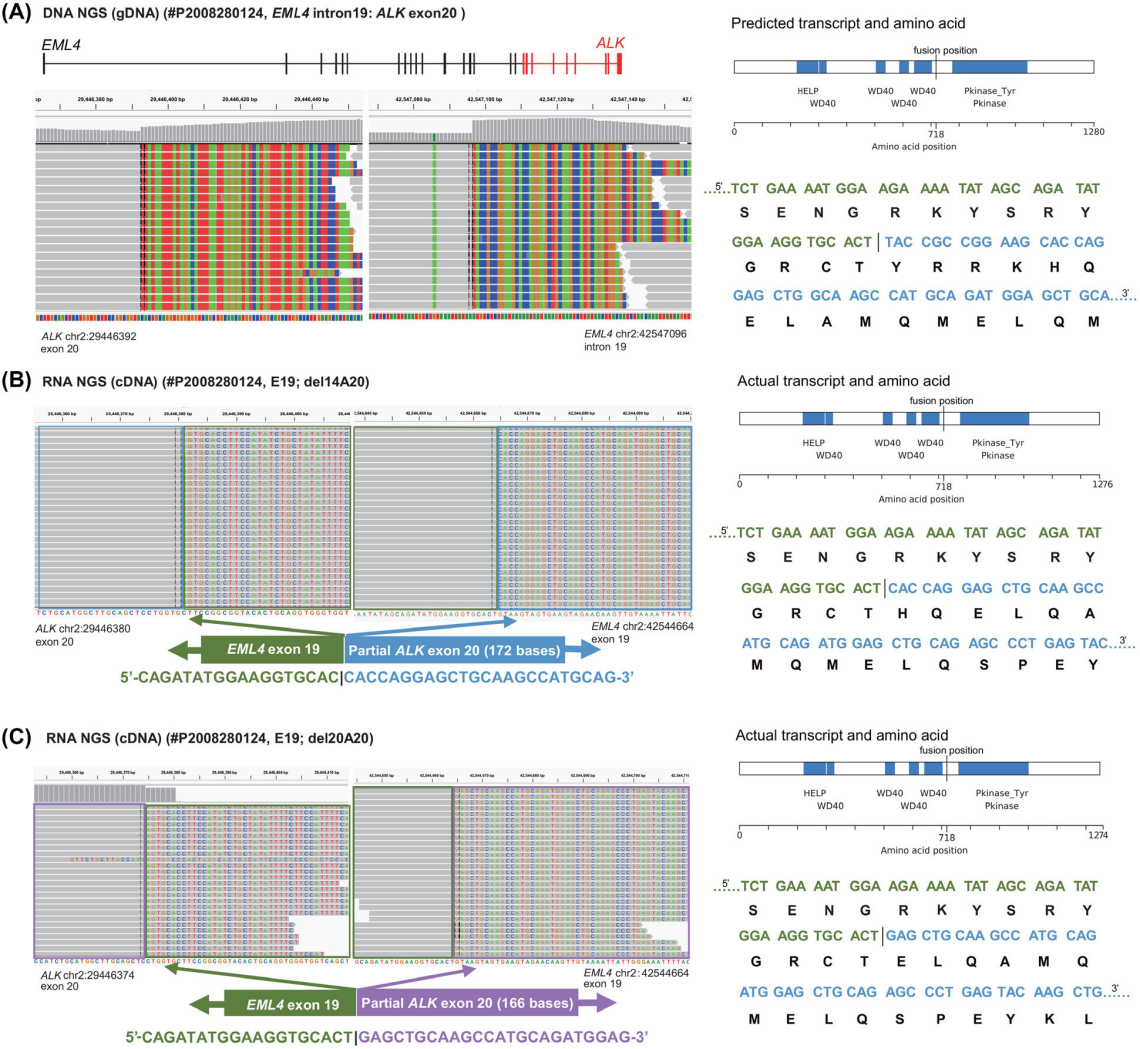
IGV screenshots, predicted or actual transcript and the amino acid sequences of *EML4‐ALK* (case #P2008280124) detected using DNA‐based NGS and RNA‐based NGS. Blue, green, red and orange blocks represent the ‘C’, ‘A’, ‘T’ and ‘G’ bases, respectively. (A) DNA‐NGS IGV screenshot of the predicted transcript and amino acid sequences. (B, C) RNA‐NGS IGV screenshots and the actual transcript and amino acid sequences of two different *EML4‐ALK* fusion types.

For canonical *EML4‐ALK* types, three cases (#P2006160041, #P2107150201 and #P1911070042) showed genomic breakpoints located in exons of *EML4* (exon 3, exon 14 and exon 21), yet the actual transcript fusion sites were altered to exon 2, exon 13, and exon 20, respectively (Fig. S4A–C). The residual exons of these three cases were not retained, and the fusion sites were skipped to adjacent exons. Thus, these three variants were classified into the known variants V1 (E13; A20), V2 (E20; A20) and V5a (E2; A20). In two additional cases (#P2010230040 and #P2009100057), the genomic breakpoints were located at exon 19 of *ALK*, but the residual exon 19 was not retained in the actual transcripts and it skipped, fusing to the boundary of exon 20 (Fig. S4D,E). These two variants were classified into the known V3b (E6ins33; A20) and V1 (E13; A20) variants. In contrast, the genomic breakpoints of another case (#P2008280124) occurred in the region of exon 20 (Fig. 4A). The actual transcript revealed that partial exon 20 was not removed, thereby retaining 172 nucleotides (14 nucleotides deleted) or 166 nucleotides (20 nucleotides deleted) of exon 20 to form multiple isoform variants with an intact kinase sequence (E19; del14A20 and E19; del20A20) (Fig. 4B,C). In addition, some of the cases with remaining tissue samples were confirmed to be positive for ALK protein expression by IHC (Fig. S2E–H). Moreover, the transcript fusion sites of two noncanonical *ALK* rearrangement‐positive cases (#P2004270003: *SQSTM1* exon 5 : *ALK* intron 19; #P2003200071: *SFTPB* exon 2 : *ALK* exon 17), whose genomic breakpoints were located in exons, were skipped to adjacent exons (*SQSTM1* exon 4 : *ALK* exon 20; *SFTPB* exon 1 : *ALK* exon 18), similar to the common exon skipping mode of *EML4‐ALK* (Table 4; Fig. S3D,E). In addition, the genomic breakpoint of *PRR23C* was out of the coding sequence (located at the 5′UTR) in case #P2009120038 (*PRR23C* exon 1 : *ALK* intron 19), and the transcript changed to *KIF5B* exon 17 : *ALK* exon 20 (Table 5; Fig. S3F).

## 4 Discussion

In this study, a total of 731 NSCLC cases with canonical *EML4‐ALK* rearrangements and 52 NSCLC cases with noncanonical *ALK* rearrangements were identified. Among them, complex genomic breakpoints of *ALK* rearrangements were detected in the exons or introns of *ALK* and its partner genes. For rearrangements whose genomic breakpoints are located in exons, their transcripts cannot be inferred from conventional splicing signals. There are also some rearrangements that result in a frameshift transcript that cannot be translated into a fusion protein containing the amino acid sequence of ALK. Therefore, the actual transcripts of these *ALK* rearrangement types were verified by RNA‐based NGS.

Frameshift of the fusion gene caused by chromosomal rearrangement is uncommon, especially in common carcinogenic‐driven fusion mutations [31]. In this study, partial canonical and noncanonical *ALK* rearrangement‐positive cases were speculated to exhibit frameshift possibilities based on DNA‐based NGS data. For the canonical *ALK* rearrangements, the results of the actual transcripts were negative in two cases (#P2008100038 and #P1902170006) and positive in the other two cases (#P2106140203 and #P2011280013). To our knowledge, reports on *ALK* fusion frameshifts are rare, and only one case has been reported in detail. In this case, *CMTR1‐ALK* (intron 2 : intron 19) was determined to be positive by DNA‐based NGS, yet the patient did not respond to crizotinib treatment, and the expression of the ALK protein was negative by IHC [32]. Presumably, the two cases in this study with genomic‐positive and transcript‐negative *EML4‐ALK* rearrangements will also not show a clinical response to ALK‐targeted inhibitors. In addition, the insertion of diverse nucleotide sequences between the nearest fusion exons (#P2106140203 and #P2011280013) prevents frame shifts and maintains the functional transcription of *EML4‐ALK* fusions, which is similar to the *EML4‐ALK* variants reported in previous studies [33, 34, 35, 36, 37, 38, 39]. Alternative splicing caused by translocation can explain inserted or deleted nucleotide sequences to maintain a multiple of 3 required for a codon in frame to produce a functional protein. In contrast, transcripts of the canonical *EML4‐ALK* fusions were detected in frame‐shift cases with noncanonical *ALK* rearrangements (#P2007070051, #P2005010014 and #L‐2018‐00005429), which are associated with a complex mechanism of chromothripsis, resulting in posttranscriptional removal of other gene sequences that joined between *ALK* and *EML4* [15, 40]. Similarly, the transformation of *PRR23C* to *KIF5B* in case #P2009120038 may also be related to chromothripsis. Therefore, the results of complex genomic rearrangement events detected by DNA‐based NGS may inaccurately reflect clinically actionable fusions [40]. Although our results showed the rarity of the predicted frameshift transcript of the *ALK* rearrangement pattern, further verification of these samples by RNA or protein assays is necessary to accurately diagnose patients at the molecular level who are candidates for targeted drug treatments.

Most genomic breakpoints of the rearranged gene occur in intronic sequences rather than in coding sequences [41]. According to conventional splicing principles, 5.6% of the rearrangement breakpoints were located in exonic regions of *ALK* or its partner genes in this study, and their predicted transcripts may be inaccurate or out‐of‐frame. Comparing the results from DNA‐based NGS and RNA‐based NGS, we found that exon skipping existed in ‘exon breakpoints’ cases carrying canonical or noncanonical *ALK* fusions. It may be reasoned that the lack of classical 3′ or 5′ accepter splice sites in the ‘exon–intron’, ‘intron–exon’ or ‘exon–exon’ structures resulted in the removal of the broken exon together with the previous intron to restore the reading frame [18, 42]. Notably, although lacking the 5′ acceptor splice site of *ALK* exon 20, the actual transcript of case #P2008280124 excluded partial nucleotides and retained a portion of exon 20 through an alternative splicing signal at the RNA level rather than implementing exon skipping splicing and it resulted in two different variants (E19; del14A20 and E19; del20A20). Further comparative analysis of the transcripts and the amino acid sequences showed that partial retention of exon 20 could ensure the in‐frame sequence with integrity of the ALK kinase domain [43, 44]. Patients harbouring multiple *EML4‐ALK* variants implied a poor prognosis due to the high heterogeneity in the tumour tissue [45]. Therefore, RNA‐based NGS showed an advantage in detecting fusion patterns in which multiple variants coexist, and more precise splicing results at the transcription level were illustrated.

However, there are some limitations of this study. Due to the retrospective nature of this study, only a small number of tissue samples were available and met the quality control necessary for RNA sequencing, so only a few samples were verified by RNA sequencing. And for some patients with advanced lung cancer, their tissue samples could not be obtained, so blood samples were taken for DNA sequencing. Furthermore, the response of some patients with these uncommon *ALK* rearrangements to ALK inhibitors is unknown, large‐scale validation of relationship between uncommon *ALK* rearrangements and treatments is necessary. In addition, the DNA NGS panel covers intronic regions where *ALK* rearrangements frequently occur and it may miss some rare intronic breakpoints. In the future, we will conduct more comprehensive clinical trials to explore the clinical benefits of these patients with specific *ALK* rearrangements from ALK inhibitor therapy.

## 5 Conclusions

In conclusion, by systematically analysing the DNA‐based NGS data of *ALK* rearrangements in lung cancer patients, we identified variable and uncommon genomic breakpoints of *ALK* and its 5′partner genes. We further verified this finding by RNA‐based NGS and found that the genomic breakpoints at the transcript level did not match those predicted by the genomic breakpoints; furthermore, we found that some of the fusions identified at the DNA level may be a false‐positive. The *ALK* fusion results at the transcript level were better able to explain their functional significance. Therefore, the identification of *ALK* fusion status in NSCLC patients may need to use orthogonal assays based on multiomics for fusion detection to achieve an accurate molecular diagnosis and ensure the reliability of the targeted drug use indicators.

## Conflict of interest

The authors declare no conflict of interest.

## Author contributions

TM, WW, ZW and YH were involved in conception and design; HT, WL and JM were involved in administrative support; Zhifang Liu, Zhengchuang Liu, HT, JM and WL were involved in the provision of study materials or patients; Zhifang Liu, YH, MX, WW, WL and HT were involved in the collection and assembly of data; ZW, YH and JZ were involved in data analysis and interpretation; all authors were involved in manuscript writing and revising and final approval of manuscript.

### Peer Review

The peer review history for this article is available at https://publons.com/publon/10.1002/1878‐0261.13348.

## Supporting information


**Fig. S1.** Integrative Genomics Viewer (IGV) screenshot of the *EML4‐ALK* fusions (#P2008100038 and #P1902170006) detected by NGS.

**Fig. S2.** IHC analysis of tissues from lung cancer patients.

**Fig. S3.** Integrative Genomics Viewer (IGV) screenshot of noncanonical *ALK* rearrangements/fusions detected by NGS (DNA‐based and RNA‐based).

**Fig. S4.** Integrative Genomics Viewer (IGV) screenshot of *EML4‐ALK* rearrangements/fusions (exon breakpoints) detected by NGS (DNA‐based and RNA‐based).

**Table S1.** DNA‐based NGS data of uncommon *ALK* rearrangements.

**Table S2.** RNA‐based NGS data of uncommon *ALK* fusions.

## Data Availability

Data are available upon request.
