# Supplementary material for: Molecular characterization of genomic breakpoints of ALK rearrangements in non‐small cell lung cancer
Source: Mol Oncol. 2022 Dec 13;17(5):765–78. doi: 10.1002/1878-0261.13348 (PMC10158786; doi:10.1002/1878-0261.13348)
Supplement: Supplementary file 1 — Fig. S1. Integrative Genomics Viewer (IGV) screenshot of the EML4‐ALK fusions (#P2008100038 and #P1902170006) detected by NGS. [file MOL2-17-765-s004.docx]

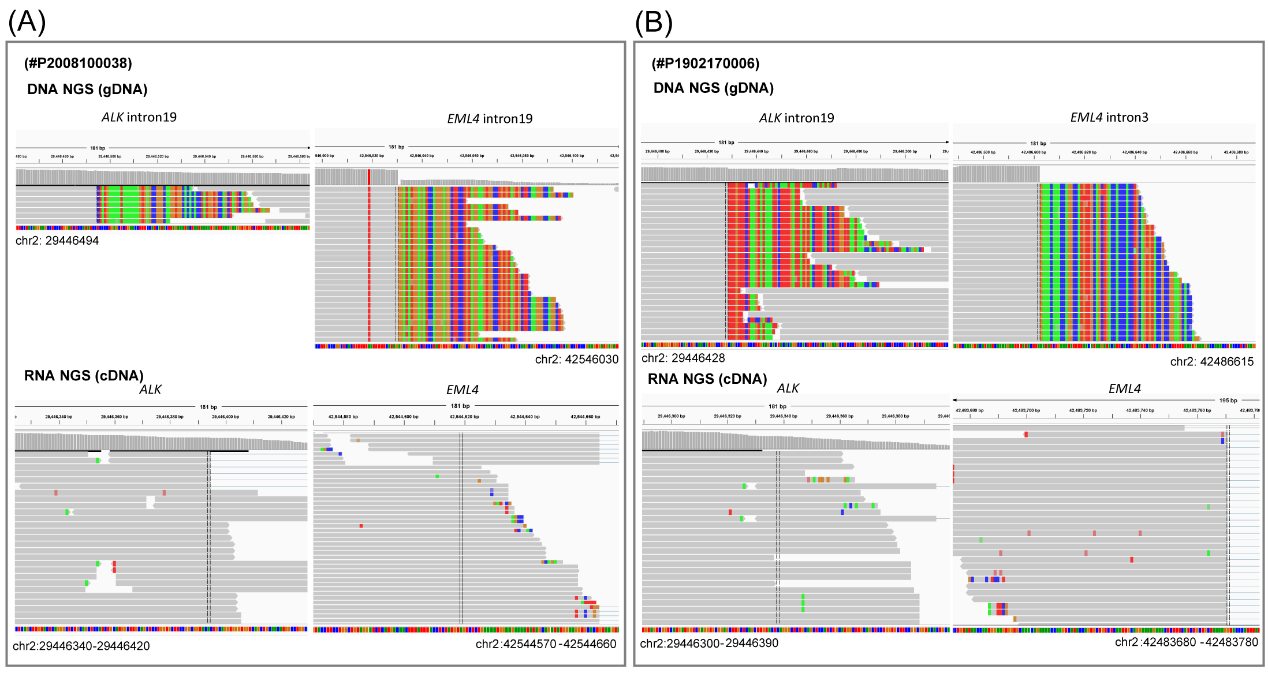


Fig. S1 Integrative Genomics Viewer (IGV) screenshot of the *EML4-ALK* fusions (#P2008100038 and #P1902170006) detected by NGS (DNA-based and RNA-based). Blue, green, red and orange blocks represent the “C”, “A”, “T”, and “G” bases, respectively. (A) The IGV DNA of case #P2008100038 shows that intron 19 of *EML4* was fused to intron 19 of *ALK*, and the IGV RNA showed fusion negativity. (B) The IGV DNA of case #P1902170006 showed that intron 3 of *EML4* was fused to intron 19 of *ALK*, and the IGV RNA showed fusion-negative results.
