# Supplementary material for: Molecular characterization of genomic breakpoints of ALK rearrangements in non‐small cell lung cancer
Source: Mol Oncol. 2022 Dec 13;17(5):765–78. doi: 10.1002/1878-0261.13348 (PMC10158786; doi:10.1002/1878-0261.13348)
Supplement: Supplementary file 2 — Fig. S2. IHC analysis of tissues from lung cancer patients. [file MOL2-17-765-s005.docx]

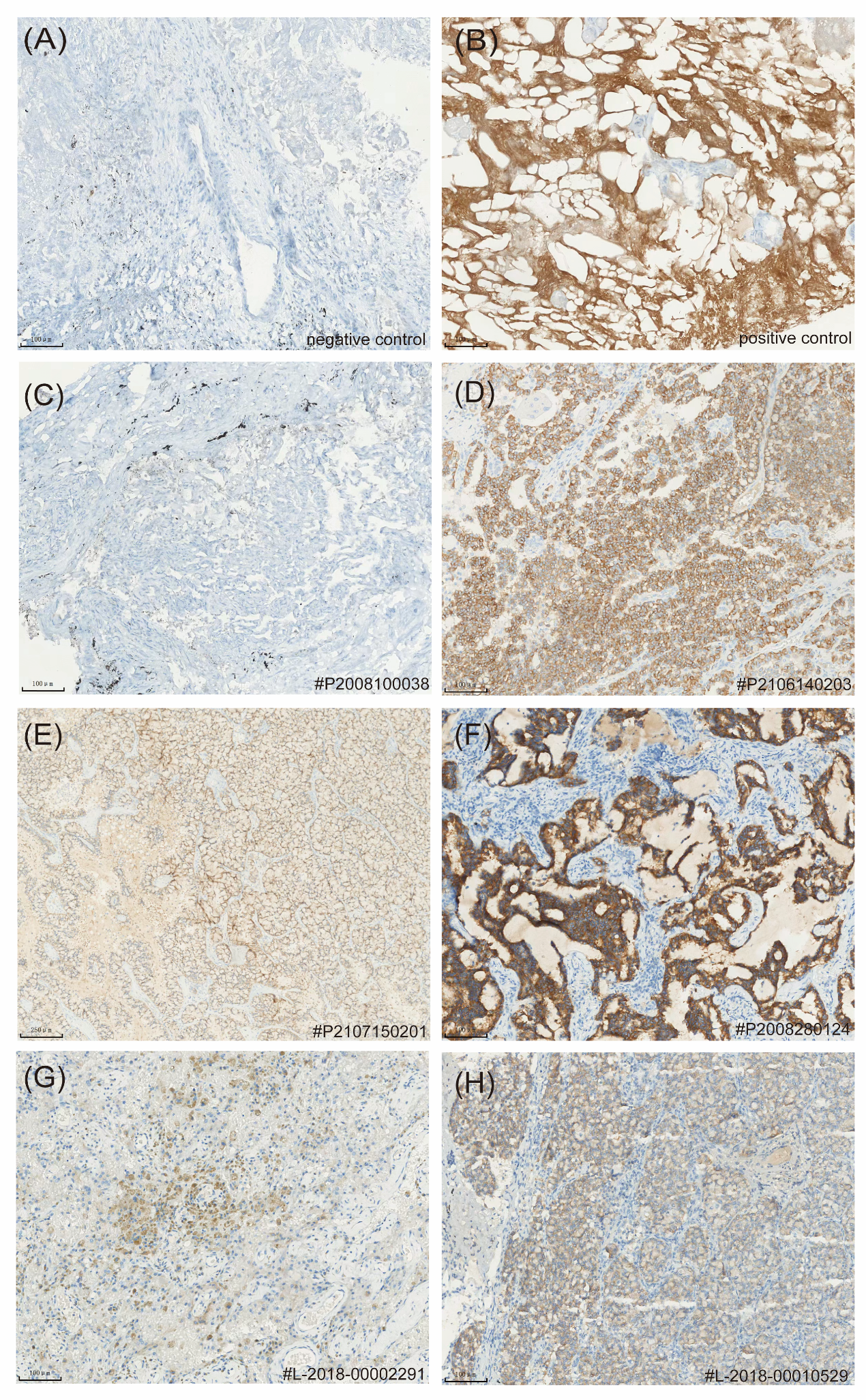


Fig. S2 IHC analysis of tissues from lung cancer patients. (A) Control examples of IHC analysis of *ALK* fusion-negative cells. Scale bar is 100 μm. (B) Control examples of IHC analysis of *ALK* fusion-positive cells. Scale bar is 100 μm. (C) Case #P2008100038, cytoplasmic staining negative. Scale bar is 100 μm. (D) Case #P2106140203 with moderate cytoplasmic staining (positive). Scale bar is 100 μm. (E) Case #P2107150201 with moderate cytoplasmic staining (positive, brain tissue). Scale bar is 250 μm. (F) Case #P2008280124 with strong cytoplasmic staining (positive). Scale bar is 100 μm. (G) Case #L-2018-00002291 with moderate cytoplasmic staining (positive). Scale bar is 100 μm. (H) Case #L-2018-00010529 with moderate cytoplasmic staining (positive). Scale bar is 100 μm.
