# Supplementary material for: Molecular characterization of genomic breakpoints of ALK rearrangements in non‐small cell lung cancer
Source: Mol Oncol. 2022 Dec 13;17(5):765–78. doi: 10.1002/1878-0261.13348 (PMC10158786; doi:10.1002/1878-0261.13348)
Supplement: Supplementary file 3 — Fig. S3. Integrative Genomics Viewer (IGV) screenshot of noncanonical ALK rearrangements/fusions detected by NGS (DNA‐based and RNA‐based). [file MOL2-17-765-s002.docx]

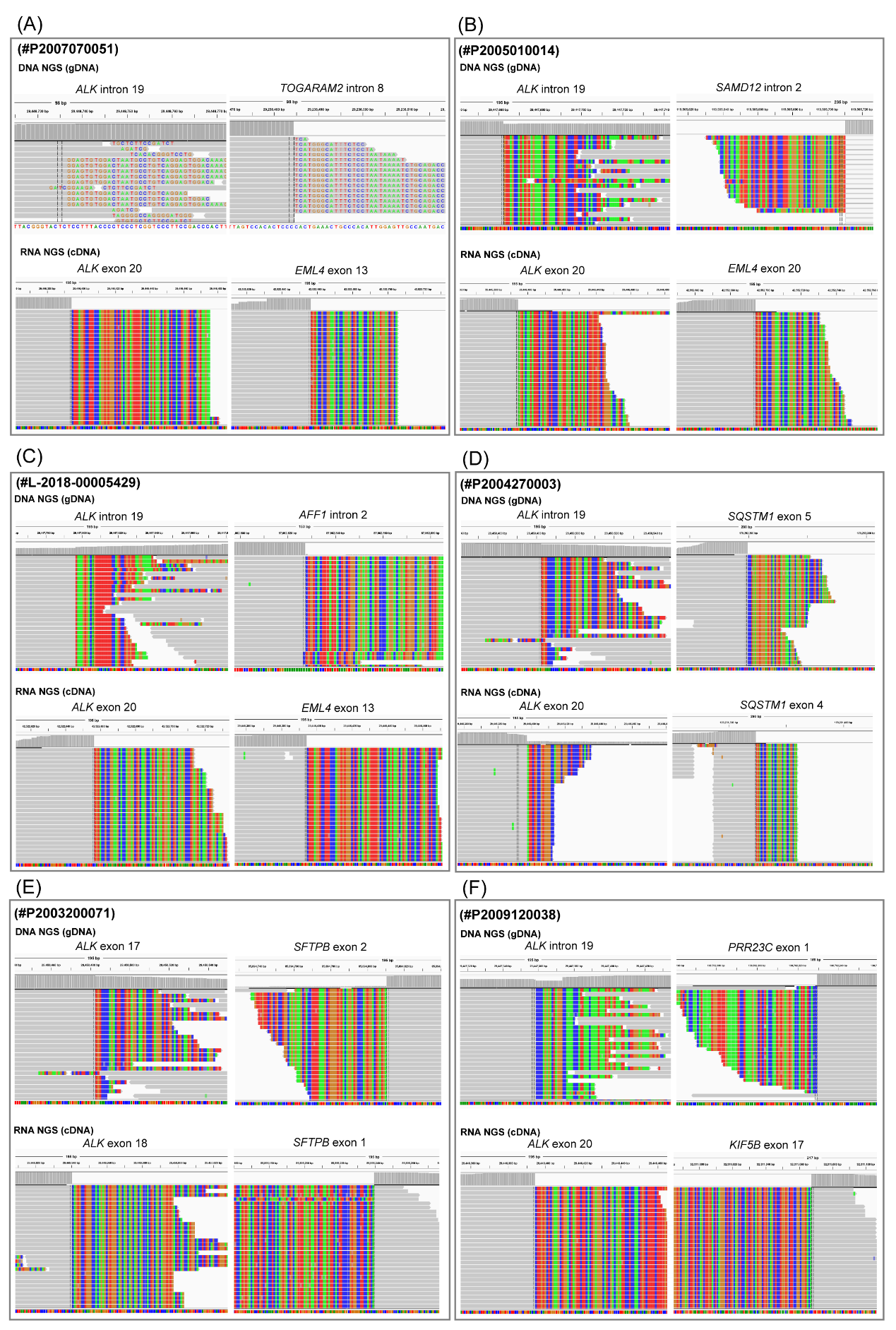


Fig. S3 Integrative Genomics Viewer (IGV) screenshot of noncanonical *ALK* rearrangements/fusions detected by NGS (DNA-based and RNA-based). Blue, green, red and orange blocks represent the “C”, “A”, “T”, and “G” bases, respectively. (A) The IGV DNA of case #P2007070051 showed intron 8 of *TOGARAM2* rearranged to intron 19 of *ALK*, and the IGV RNA showed exon 13 of *EML4* fused to exon 20 of *ALK*. (B) The IGV DNA of case #P2005010014 showed intron 2 of *SAMD12* rearranged to intron 19 of *ALK*, and the IGV RNA showed exon 20 of *EML4* fused to exon 20 of *ALK*. (C) The IGV DNA of case #L-2018-00005429 showed intron 2 of *AFF1* rearranged to intron 19 of *ALK*, and the IGV RNA showed exon 13 of *EML4* fused to exon 20 of *ALK*. (D) The IGV DNA of case #P2004270003 showed exon 5 of *SQSTM1* rearranged to intron 19 of *ALK*, and the IGV RNA showed exon 4 of *SQSTM1* fused to exon 20 of *ALK*. (E) The IGV DNA of case #P2003200071 showed exon 2 of *SFTPB* rearranged to exon 17 of *ALK*, and the IGV RNA showed exon 1 of *SFTPB* fused to exon 18 of *ALK*. (F) The IGV DNA of case #P2009120038 showed exon 1 (located at5’UTR) of *SFTPB* rearranged to intron 19 of *ALK*, and the IGV RNA showed exon 17 of *KIF5B* fused to exon 20 of *ALK*.
