# Supplementary material for: Molecular characterization of genomic breakpoints of ALK rearrangements in non‐small cell lung cancer
Source: Mol Oncol. 2022 Dec 13;17(5):765–78. doi: 10.1002/1878-0261.13348 (PMC10158786; doi:10.1002/1878-0261.13348)
Supplement: Supplementary file 4 — Fig. S4. Integrative Genomics Viewer (IGV) screenshot of EML4‐ALK rearrangements/fusions (exon breakpoints) detected by NGS (DNA‐based and RNA‐based). [file MOL2-17-765-s006.docx]

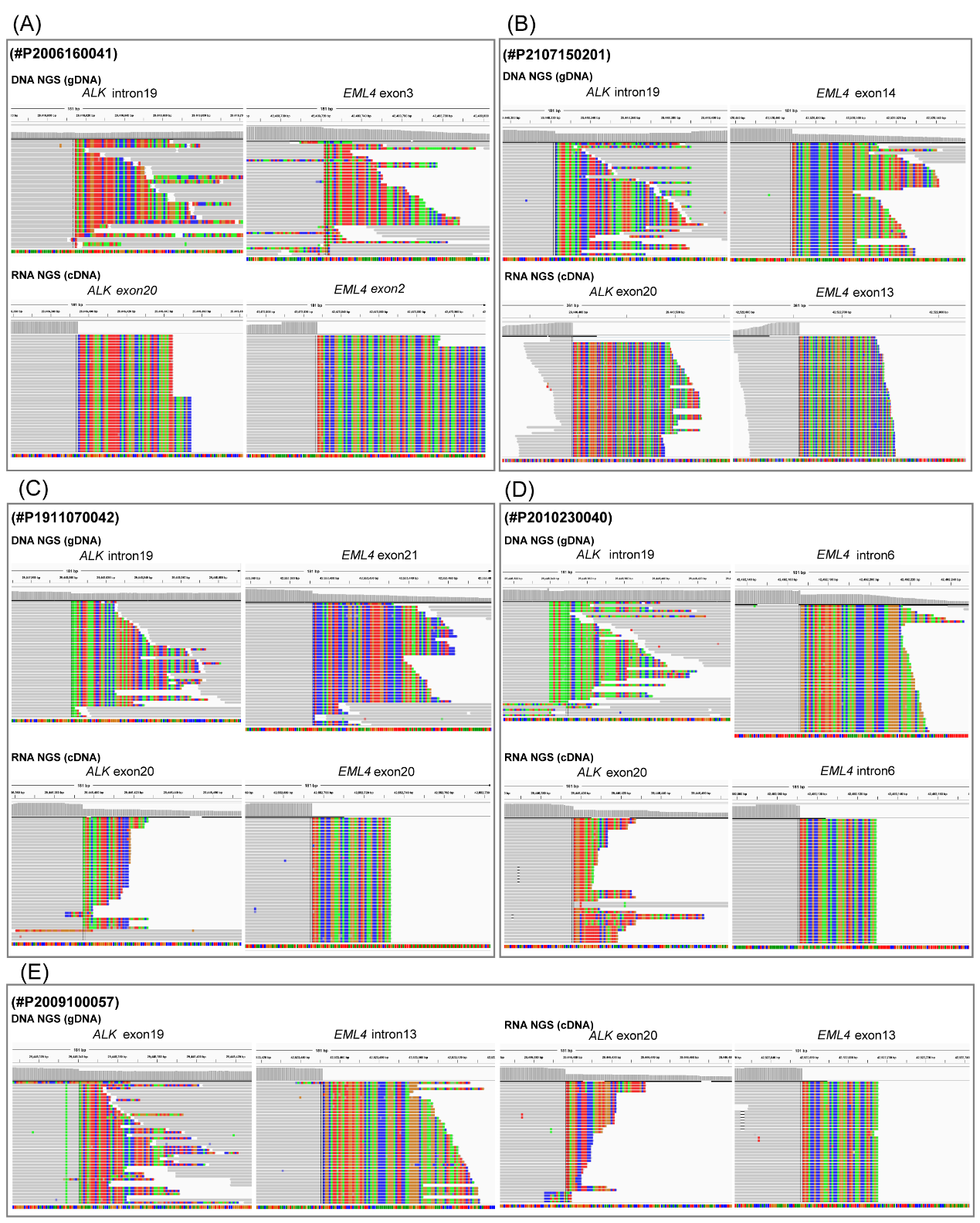


Fig. S4 Integrative Genomics Viewer (IGV) screenshot of *EML4-ALK* rearrangements/fusions (exon breakpoints) detected by NGS (DNA-based and RNA-based). Blue, green, red and orange blocks represent the “C”, “A”, “T”, and “G” bases, respectively. (A) The IGV DNA of case #P2006160041 showed exon 3 of *EML4* rearranged to intron 19 of *ALK*, and the IGV RNA showed exon 2 of *EML4* fused to exon 20 of *ALK*. (B) The IGV DNA of case #P2107150201 showed exon 14 of *EML4* rearranged to intron 19 of *ALK*, and the IGV RNA showed exon 13 of *EML4* fused to exon 20 of *ALK*. (C) The IGV DNA of case #P1911070042 showed exon 21 of *EML4* rearranged to intron 19 of *ALK*, and the IGV RNA showed exon 20 of *EML4* fused to exon 20 of *ALK*. (D) The IGV DNA of case #P2010230040 showed intron 6 of *EML4* rearranged to intron 19 of *ALK*, and the IGV RNA showed intron 6 of *EML4* fused to exon 20 of *ALK* (V3b). (E) The IGV DNA of case #P2009100057 showed intron 13 of *EML4* rearranged to intron 19 of *ALK*, and the IGV RNA showed exon 13 of *EML4* fused to exon 20 of *ALK*.
